# Evaluating the Microbial Habitability of Rogue Planets and Proposing Speculative Scenarios on How They Might Act as Vectors for Panspermia

**DOI:** 10.3390/life11080833

**Published:** 2021-08-14

**Authors:** Dirk Schulze-Makuch, Alberto G. Fairén

**Affiliations:** 1 Astrobiology Group, Center for Astronomy and Astrophysics, Technische Universität Berlin, 10623 Berlin, Germany; 2 GFZ German Research Center for Geosciences, Section Geomicrobiology, 14473 Potsdam, Germany; 3 Department of Experimental Limnology, Leibniz-Institute of Freshwater Ecology and Inland Fisheries (IGB), 16775 Stechlin, Germany; 4 School of the Environment, Washington State University, Pullman, WA 99163, USA; 5 Centro de Astrobiología (CSIC-INTA), 28850 Madrid, Spain; agfairen@cab.inta-csic.es; 6 Department of Astronomy, Cornell University, Ithaca, NY 14853, USA

**Keywords:** rogue planet, microbial life, habitability, panspermia

## Abstract

There are two types of rogue planets, sub-brown dwarfs and “rocky” rogue planets. Sub-brown dwarfs are unlikely to be habitable or even host life, but rocky rogue planets may have a liquid ocean under a thick atmosphere or an ice layer. If they are overlain by an insulating ice layer, they are also referred to as Steppenwolf planets. However, given the poor detectability of rocky rogue planets, there is still no direct evidence of the presence of water or ice on them. Here we discuss the possibility that these types of rogue planets could harbor unicellular organisms, conceivably based on a variety of different energy sources, including chemical, osmotic, thermal, and luminous energy. Further, given the theoretically predicted high number of rogue planets in the galaxy, we speculate that rogue planets could serve as a source for galactic panspermia, transferring life to other planetary systems.

## 1. Introduction

One of the implicit assumptions in the search for life is that life must evolve on a planet that orbits a star. The Drake equation [1] exemplifies this assumption, not accounting for any life that could exist on a rogue, or interstellar, planet. Indeed, energy is a prerequisite for life [2], and the most obvious source of energy is the light from a star [3]. However, rogue planets may not be as desolate and barren as generally thought. There are two types of rogue planets, with very different environments.

The first type of rogue planet are failed stars, not nearly massive enough for nuclear fusion to ignite their cores. These so-called “sub-brown dwarfs” [4] would be similar in composition to the gas giants that we know of within our solar system. Their sizes could range from several times the mass of Jupiter to as small as Uranus [5]. Like Jupiter, radiation levels would be very high, and there would be a seamless transition from gas to liquid in their deep atmospheres. Convective zones would mix the inner atmosphere with the outer surface, bringing any organic compounds on the surface down into extreme temperatures and pressures. Life within a gas giant has to be considered extremely unlikely. Although gas giants may contain liquid water and even organic compounds in their atmosphere, like Neptune in our solar system [6], an origin of life in an atmosphere, not involving a solid-liquid interface, is difficult to imagine, as is a transfer of life from a rocky planet or icy moon to a gas giant [7]. However, that type of rogue planet may be accompanied by a large moon, which could be a potential abode of life. The first rogue planets of this type have been detected in 2012 and 2013 and have been estimated to have several times the mass of Jupiter [8,9].

The second way a rogue planet may form is from gravitational dynamics in an early solar system: small “rocky” planets get too close to very large planets in the chaotic movements before the orbits are stabilized [10]. These encounters could potentially cast the smaller planet out of its native solar system, letting it wander the galaxy as a rogue planet [11]. If a planet is ejected from a solar system during planetary formation, it could be very similar to Earth or the other inner planets or asteroids. It could have a sufficient inventory of radioactive elements to heat and also melt its interior [12]. Life could have originated on such an Earth-type planet before it was ejected from its solar system, especially if it had a reducing atmosphere. In 2020 the first terrestrial-mass rogue planet candidate was claimed to be detected using microlensing [13], showing that Mars to Earth-sized mass rogue planets can be detected and characterized in principle using current technology. However, detections are extremely challenging, because Mars to Earth-sized planets are already difficult to identify when in orbit around a star. However, on theoretical grounds, many of these terrestrial mass rogue planets should exist [13]. In the following, we focus on the possibilities for life in these terrestrial types of rogue planets.

## 2. Liquid Water on Rogue Planets

**Figure 1:**
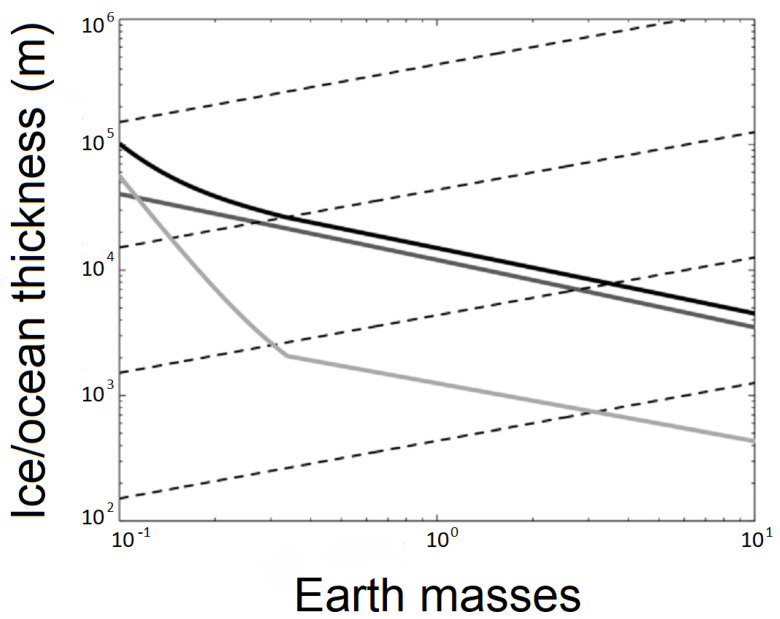
Amount of ice necessary to create a liquid ocean on a Steppenwolf planet (modified from [20]).

Liquid water is a prerequisite for life as we know it (e.g., [14]). Given the poor detectability of rogue planets, there is still no direct measurement evidence of the presence of water on them, although several hypotheses have been considered in the past regarding the presence of water within rogue planets. Once ejected, any possible atmosphere of a rogue planet would collapse and freeze onto its surface. However, it has been suggested that heat from radioactivity in the core could provide sufficient energy to keep the water liquid beneath the newly formed icy surface [12,15]. This could be enough energy to sustain a liquid ocean of water, provided it is insulated by a layer of ice on the outer surface (Figure 1). Lingam and Loeb [16,17] estimated that planets with a subsurface ocean could be 100 to 1000 times more common than rocky planets in the habitable zone of stars, with many of these planets having the potential of being transformed into rogue planets. Since water is such a common compound in the universe (given also that hydrogen is the most common element and oxygen the third-most common element) [7], it seems reasonable that many of these icy worlds may have ice caps consisting dominantly of water ice with liquid water beneath, as also inferred for the icy moons Europa, Enceladus, and Ganymede (likely among others) in our own solar system. It has been estimated that the maximum biomass that might be sustainable in the deep subsurface of that type of environment would be a few percent of that of Earth’s biosphere based on energetic considerations [18], which is roughly comparable to earlier modeling studies of the European subsurface ocean [19].

A rogue planet that is harboring a subglacial ocean has been termed a Steppenwolf planet because any life in that type of habitat would be analogous to a lone wolf wandering the galactic steppe [20]. The lines in Figure 1 show how much ice is necessary to sustain a liquid ocean underneath as a function of the mass of the planet. The black line represents the most realistic scenario, that of geothermal heat flux into the ocean equaling the radiative heat flux out of it. The gray line represents a mixture of water and ammonia. The light gray line represents an unlikely scenario where carbon dioxide vents push their gas up to the surface, where it freezes and falls to the ground to form a very insulating layer.

Furthermore, under certain conditions, an ice layer might not even be necessary. It has been suggested that a thick atmosphere of molecular hydrogen can keep enough pressure and provide enough insulation to have liquid water on the outer surface of the planet without needing an ice layer. This atmosphere will have to be 10^2^–10^4^ bars [21]. If the planet would be ejected out of the solar system quickly, enough hydrogen may be retained for such a thick atmosphere and not being lost into space. Interestingly, if Earth were cast out of the solar system. It would not have the required amount of water–it would take a planet with similar composition, but 3.5 times more mass than Earth [20]. Alternatively, Badescu [22] suggested that an optically thick atmosphere of methane, ethane, and carbon dioxide could also enable water, ammonia, or ethane oceans on a rogue planet. We feel that this option is most interesting for solvents with a low liquidity temperature such as mixtures of ammonia and water, or ethane, and if life as we don’t know it can exist in these solvents. The moon Titan in our solar system may be an ideal planetary body to closely analyze exactly this possibility [23]. One problem with the envisioned thick atmospheres [22] is that it may be difficult for a rogue planet to retain a sufficiently thick atmosphere for timescales of possibly billions of years. However, the problem can be overcome if the rogue planet has elevated radionuclide substances, which–if sufficiently high-may allow the presence of non-aqueous liquids on the surface of that planet, even if it has only a thin atmosphere [24].

## 3. Energy Sources for Life within Rogue Planets

### 3.1. Chemical Energy

The most basic energy source for life is chemical energy, and it has been proposed also as the first form of energy used by life on Earth [7], mostly because it doesn´t require a conversion from different energy sources. It is unclear which metabolic pathway was the first on our planet. A basic autotrophic pathway seems to be methanogenesis [25], which could be utilized nearly anywhere where volcanic activity is present, converting carbon dioxide and hydrogen gas into water and methane. Alternatively, heterotrophy has been suggested as the first metabolic pathway on Earth [26,27], which may have used organic macromolecules supplied by prebiotic synthesis [28] or cometary delivery [29]. Chemical energy would also be an obvious possibility for life on a rogue planet. There are subsurface lithoautotrophic microbial ecosystems on Earth, which are not dependent on surface-based photosynthesis, including methanogens, homoacetogens, and sulfate-reducers, all mainly strictly anaerobes [30]. Metabolic activity has been measured in subseafloor sediments, continental flood basalts, and granitic plutons, orders of magnitude lower than that of life on Earth’s surface [30,31]. In these environments, autotrophic microorganisms outnumber heterotrophs [32]. Gold [33] argued that the terrestrial deep biosphere could host a total biomass equivalent or even larger than that at the Earth’s surface. Importantly for rogue planets, the subsurface realm would be a highly stable environment that may safeguard inhabitants against extremes and radical changes [31,34]. And there is evidence that chemolithotrophic metabolism is viable using iron meteorites as the sole source of energy [35]. Therefore, whole rogue planets, beyond just meteorite pieces, should provide a viable and stable environment as a habitat for microorganisms during interstellar voyages.

### 3.2. Light Energy and Photosynthesis

Almost every form of life on Earth depends directly or indirectly on photosynthesis, so it is challenging to think of an evolutionary pathway in which photosynthesis does not appear. No nearby star to a rogue planet means that photosynthesis will face challenges to develop as a common means of energy. One possibility in rogue planets is photosynthesis independent of the light from a star. On Earth, some bacteria do photosynthesis from the extremely feeble near-infrared glow of hydrothermal vents at the bottom of the ocean, where no sunlight reaches [36]. We do not know if photosynthesis on Earth first appeared on the surface and whether these seafloor bacteria have adapted later to the feeble glow, or if it was the other way around. It is also conceivable that photosynthetic microorganisms of an ice-covered ocean within a rogue planet could take advantage of surface sunlight during the brief periods of transit near a star, briefly flourishing near the surface with increased metabolic activity, to hide again in the dark subsurface for eons looking for the feeble glow of the vents to survive until another star comes close. This is a scenario difficult to envision in our part of the galaxy, which is quite remote but may be more likely close to the center of the galaxy with a much higher frequency of stars.

### 3.3. Thermal Energy and Thermosynthesis

There are two other possible, maybe more intriguing energy sources on a rogue planet. Hydrothermal vents on the ocean bottom are used by life on Earth for metabolism via redox reactions, but in principle, thermotropic life forms that may exist in the subsurface oceans may harvest the energy provided from hydrothermal vents by using thermal gradients or heat directly. Muller [37,38,39] and Muller and Schulze-Makuch [40] suggested the use of thermal gradients, which they termed thermosynthesis, as a plausible metabolic pathway. A steam engine makes use of a phase transition and so could thermosynthesis. The mobility of the molecules within a membrane would increase when membranes are undergoing the thermotropic phase transition [40]. This kind of transition could plausibly result in a change in potential across the membrane due to a change in dipole potential [39]. Very similar potential changes that undergo the thermotropic phase transition have been measured across monolayers of lipids at the water/air interface [7]. As these changes can easily reach 100 mV, they would be high enough to drive ATP synthesis [40]. If this reasoning is correct, thermosynthesis could also be a basic pathway of metabolism for organisms on early Earth, possibly a progenitor of bacterial photosynthesis [38,39]. In addition, it has also been considered as an option for possible life in Europa’s subsurface ocean [41].

**Figure 2:**
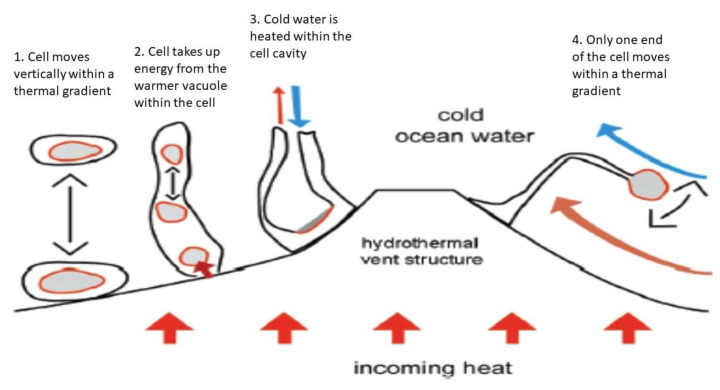
A sketch of how various types of thermotrophs could gain energy near a hydrothermal vent structure. In gray are marked the hotter areas within the model organism from which thermal energy can be obtained. In model (1), the thermotroph moves off the vent structure, but the vacuole is hotter and the putative organism can obtain energy from the thermal gradient. The principle is the same for (2) to (4), but the model organisms remain attached to the substrate. The heated vacuole moves within the cell (2), colder circulating water is heated within the organism (3), or an appendix of the cell moves in the thermal gradient back and forth (4) (modified from [40]).

Alternatively, “thermotrophs” could harvest energy from the high heat capacity of water, which is about 4 kJ/kg under a wide range of temperature and pressure conditions [7]. If a cell mass of 10^−12^ g is assumed, which is similar to microbes on Earth [42], and if we further assume that one-tenth of the total cell mass is a water-filled vacuole, then the thermotropic organism could gain about 2.5 × 10^6^ eV of energy by lowering the temperature of the vacuole by 1 K. The organism could extract about 9000 eV of usable energy for a temperature change, for example, from 278 K to 277 K using the Carnot cycle [7,41]. That energy gain could be further increased if a temperature differential of more than 1 K is tapped. In principle, the vacuole, which is filled with hot water, would function as an internal heat engine and at the same time may be used to provide the organism with the needed buoyancy to float in the ocean water (Figure 2). One way the temperature gradient between the vacuole and the cell plasma could be used is to produce high-energy metabolites via conformational changes. If a cell is as large as the giant pantropical alga, *Valonia macrophysa* [43], which has a water vacuole of about 10 g, the potential energy yield that could be extracted is close to 6.2 × 10^18^ eV or 1 Joule [7].

Inefficiency is a potential drawback to the use of thermal energy because the most efficient thermodynamic system known is the Carnot cycle. Thus, most of the energy in a thermal gradient would be dissipated as heat without being captured by chemical bonds, and would also degrade the thermal gradient itself. A possible adaptation by the thermotropic organism would be to shuttle back and forth across fairly sharp temperature gradients or to possess an elongated body and make use of convection to dissipate the unusable entropy-related energy (Figure 2).

### 3.4. Osmotic Energy

An even more speculative source of energy would be energy obtained from osmotic gradients. No organism on Earth is known to use osmotic gradients as an energy source, but this could simply be the case because light and chemical energy are readily available on our planet and also provides a larger amount of energy. If life originated on a rogue planet early in its history, it would have been exposed to significant salinity gradients given that large water reservoirs would have not been yet in chemical equilibrium with the rocky mantle [41]. Microbial life may have remained in a hypo-osmotic cellular state even as the liquid water reservoirs became progressively saltier. Various organisms on Earth can withstand high osmotic gradients. One example is teleosts, which are bony fish such as sharks. They originated in fresh water and retained an osmotic differential of about 0.7 osmoles between their intercellular fluids and the surrounding environment [44]. Some halophilic organisms can tolerate much higher gradients [45]. For example, the yeast *D. hansenii* can be found in hypersaline environments like the Great Salt Lake of Utah and has been shown to grow in media containing up to 4 M NaCl [46]. Thus, it seems plausible that an organism adapted to use salinity gradients as an energy source could use an osmotic differential of at least five times the value that sharks are adapted to.

Using a value of 3.5 osmoles as a first conservative assumption, we can calculate the energy yield as detailed in Schulze-Makuch and Irwin [7]: briefly, the osmotic pressure is calculated by multiplying the molar solute concentration with the universal gas constant and the absolute temperature. Then the osmotic pressure is multiplied by the cross-sectional area of one water molecule to determine the force that acts upon one water molecule along its concentration gradient. Finally, the energy gain is determined by multiplying the calculated force by the distance the water molecule moves down its density gradient. If we assume the distance to be 10^−8^ m for a biomembrane, then the calculated potential energy yield is 0.035 eV. Putting this in context with life on Earth as we know it, one ATP can be phosphorylated from ADP for every 9 water molecules entering the cell by osmosis. This is certainly much less efficient than chemical or light energy, where the energy yield per reaction is around 2 eV, depending on the frequency of the light and the specific redox-reaction considered, respectively. The direct coupling of the movement of water molecules to phosphorylation reactions is not known for life on our planet, but it could be an option when other energy sources are not as readily available.

Schulze-Makuch and Irwin [41] suggested as a plausible mechanism tertiary structural changes in a channel-associated protein that would catalyze the formation of high energy bonds, similar to ligand-induced conformational changes in membrane receptors that lead to a series of steps culminating in the synthesis of high-energy cyclic AMP. Alternatively, a reaction to form a high-energy bond could occur inside the cell when water is moving inward along a membrane water channel from the hypotonic surrounding environment or in the opposite direction when the water leaves the cell toward its hypertonic surrounding environment. If so, then a putative organism could move between different layers of salinity and each way being able to harvest energy. Enough energy could be in principle gained to support an ecosystem [19,47].

### 3.5. Other Potential Energy Sources

Many other energy sources could in principle be utilized by life. However, those are typically too low in energy gain or are too unpredictable to be used, for example, magnetic fields, gravitational energy, radiolytic reactions, or electron oxidation. The energy from magnetic fields could in principle be harvested by microorganisms via the Lorentz force or via induction, but our planet´s magnetic field is not strong enough to make this a competitive energy source compared to chemical or light energy [7]. However, magnetotactic bacteria [48], and even some animals [49], are sensitive to magnetic fields, and some planets are exposed to much stronger magnetic fields than Earth, especially those close to neutron stars or magnetars [50]. Thus, magnetic fields are an energy source that could be harvested on some extraterrestrial worlds. In the case of gravitational/tidal energy, if a rogue planet is ejected together with its moon, which has been estimated to not be a rare occurrence [51], then tidal energy would also be available to produce additional heating. Another source of energy could be radioactivity: although it provides in principle ample amounts of energy, the random fashion of the associated atomic decay and bursts of energy when it occurs may prove too difficult to control for an organism [7]. Nevertheless, radioactivity is largely responsible for the heating of the interior of a planetary body, which then could be utilized by life as outlined above under Section 3.3. In addition, recent experimental studies of marine sediment and sedimentary minerals [52,53] have found that radiolytic H_2_ yields per unit radiation are magnified by up to 27 times relative to pure water, depending on sediment composition. And this radiolytic H_2_ is produced at all sediment depths, suggesting that water radiolysis may be the key driver of microbial activity in a broad range of settings in marine sediments on Earth older than a few million years. Finally, laboratory studies have demonstrated that electrochemical enrichment is a feasible approach for the isolation of microbes capable of gaining electrons directly from insoluble minerals [54,55], including electrochemically active sulfur-oxidizing microbes and sulfate-reducing microbes capable of cathode oxidation.

## 4. Discussion

Astronomical data on rogue planets are extremely poor at the moment. Previous estimates suggest that the total number of compact objects in the mass range 10^−8^–10^−2^ M_⊙_, unbound to a host star, could reach 100,000 per main-sequence star. From this total population of the so-called “nomads”, up to some tens can have mass enough to be considered rogue planets [56]. The vast majority, however, would be small objects with a mass below that of Mars. We may have experienced a couple of visits from such extrasolar objects recently: the rocky object ‘Oumuamua [57] and the comet 2I/Borisov [58]. It is also very likely that these encounters are more prevalent than previously thought, and that we are now recording these high incidences of visits (2 in the past 5 years vs. none in the previous 50) just as a result of the improvement of our detection systems.

Only very few rogue planets are known [8,9,13] because they are so difficult to detect. Most exoplanets are discovered through their interactions with their parent star, which clearly is not an option for rogue planets. Estimates of the number of rogue planets range between 2 and 60 rogue planets per star in our galaxy [56]. Assuming the higher bound, then statistically, a rogue planet would cross the path of the inner Solar System once every 25 million years [59].

If the ice layer of the rogue planet is convective, oxidants from the surface could be sinking to the subsurface liquid oceans and provide oxygen to any microbial life, similarly as proposed for Europa [60]. Thus, nutrient cycling could occur within a rogue planet even without plate tectonics, which is the major recycling mechanism on Earth. When considering possible life on another world, including a rogue planet, we have to be open-minded for novel adaptations of how life might have dealt with the environmental challenges at hand [47], but for a Steppenwolf-type of a rogue planet, the icy moons in our solar system may be a first suitable analog model.

The convective flow up to the outer surface of the rogue planet, as has been suggested for Europa [61], can provide a route to follow for potential microbes, which then could become eventually trapped and encased in a dormant state within the ice. Thus, rogue planets could function as a repository for dormant or fossilized life. In permanently frozen habitats on Earth, even some multicellular organisms may be preserved for tens of thousands of years, including stems of Antarctic moss successfully regrown from an over 1000-year sample covered by ice for about 400 years [62], rotifers recovered from 24,000-year-old Arctic permafrost [63], nematodes revived from the Siberian permafrost with source sediments dated over 30,000 years [64], and campion plants regenerated from seed tissue preserved in relict 32,000-year-old permafrost [65]. Of course, access to any dormant or fossilized life, set aside the technological challenge it will represent, may be difficult due to the radiation environment of interstellar space and only be found at least several meters beneath the planetary surface.

But how could a transfer of microorganisms from a rogue planet to a habitable planet occur? Lingam and Loeb [16] suggested that a rogue planet could be temporarily captured by a star, and then would seed another planet that orbits the same star. This scenario has been proposed previously for the transfer of microbial life from Mars to Earth or vice versa (e.g., [66,67,68]), so is reasonable. Lingam and Loeb [16] calculated various probabilities for this event and concluded that the chances for this type of interplanetary panspermia are raised by several orders of magnitude in a closely packed planetary system with a low-mass star. Certainly, the proximity of the rogue planet, which was temporarily captured by the star, would enhance the probabilities of transfer of microbial life from the rogue planet.

A different approach was taken by Wickramasinghe et al. [69], who pointed out that large impacts–like the one that resulted in the Chicxulub crater 65 million years ago–would eject large amounts of debris, including a microbial load, into the zodiacal cloud. During the impact, a significant fraction of material would not have been shocked and heated to sterilization levels, and microbes may be able to stay viable in the cloud for millions of years [70,71]. Wickramasinghe et al. [69] considered that scenario for an Earth-type planet seeding a rogue planet as realistic. If so, the same should be true for the reverse transport, from the rogue planet to the planet within the solar system.

In addition to the two scenarios above, given in the literature, we can envision and propose here two additional scenarios. The first one is if a rogue planet passes closely by a habitable planet, part of the outer layer of the rogue planet could be blown away by gravitational disturbances, only to be gathered up by the habitable rocky planet. Dormant life that had been trapped in the icy layer might become active again and establish a biosphere on the habitable planet. In a solar system like ours, that scenario could take place on an Earth-like planet in the inner solar system, or maybe at an icy moon of the outer solar system.

The second additional scenario is that the rogue planet may end up colliding (or undergoing a near-collision) with one of the planets in the solar system. This can be generally thought of as a sterilizing effect, such as when a Mars-sized object collided with Earth in early solar system history that resulted in the creation of the Moon [72,73]. However, given an expected heterogeneous distribution of energy and matter, it is not out of the question that some dormant life may survive that impact, and eventually seeds the planet with which the rogue planet collided. The Mars-size impactor could have, in fact, been a rogue planet (or, more likely, became one after the collision). In the case of the creation of Earth´s Moon, it seems possible that early Earth and the early Moon shared a common atmosphere. A rogue planet may also conceivably have changed the orbiting direction of Venus or stripped Mercury of most of its mantle and crust [74].

We realize that the transfer of life from a rogue planet to a habitable planet can only occur under very constrained environmental conditions. Certainly, all four scenarios for panspermia above are highly speculative. However, given current estimations of so many rogue planets and also evidence for so many major impacts—as evidenced by the cratering records on the planets and moons in our solar system—in our galaxy and also the universe, these are nevertheless mechanisms that merit further investigation and modeling to reveal which of these proposed processes may offer a realistic transfer process. However, a more in-depth treatise including modeling is beyond the objectives of this work.

## 5. Conclusions

We have presented here the hypothesis that microbial life could exist within rogue planets, especially if it originated there very early on during their natural history. Life could use a variety of energy sources, including energy sources that are not used by life on Earth. Rogue planets could also serve as a source pool for galactic panspermia given the enormous number of rogue planets estimated to exist in the galaxy and frequent impacts, even if the transfer mechanism of life may only be feasible in rare circumstances. The likelihood of a feasible life transfer mechanism should be further investigated and modeled.

## Data Availability

Not applicable.

## References

[1] Drake F.D. (1962). Intelligent Life in Space.

[2] Kok B., Radmer R., Ponnamperuma C. (1976). Energy requirements of a biosphere. Chemical Evolution of the Giant Planets.

[3] Youvan D.C., Mars B.L. (1987). Molecular mechanisms of photosynthesis. Sci. Am..

[4] Caballero J.A. (2018). A review on substellar objects below the deuterium burning mass limit: Planets, brown dwarfs or what?. Geosciences.

[5] Sengupta S., Sengupta S. (2015). Brown dwarfs: The missing link between stars and planets. Worlds Beyond Our Own.

[6] Baker V.R., Dohm J.M., Fairén A.G., Ferré T.P.A., Ferris J.C., Miyamoto H., Schulze-Makuch D. (2005). Extraterrestrial hydrogeology. Hydrogeol. J..

[7] Schulze-Makuch D., Irwin L.N. (2018). Life in the Universe: Expectations and Constraints.

[8] Delorme P., Gagné J., Malo L., Reylé C., Artigau E., Albert L., Forveille T., Delfosse X., Allard F., Homeier D. (2012). CFBDSIR2149-0403: A 4–7 Jupiter-mass free-floating planet in the young moving group AB Doradus?. Astron. Astrophys..

[9] Liu M.C., Magnier E.A., Deacon N.R., Allers K.N., Dupuy T.J., Kotson M.C., Aller K.M., Burgett W.S., Chambers K.C., Draper P.W. (2013). The extremely red, young L dwarf PSO J318.5338–22.8603: A free-floating planetary-mass analog to directly imaged young gas-giant planets. Astrophys. J. Lett..

[10] Lund M.B. (2019). Worlds in migration. arXiv.

[11] Bromley B.C., Kenyon S.J. (2014). The fate of scattered planets. Astrophys. J..

[12] Bada J.L. (2001). State-of-the-art instruments for detecting extraterrestrial life. Proc. Natl. Acad. Sci. USA.

[13] Mróz P., Poleski R., Gould A., Udalski A., Sumi T., Szymański M.K., Soszyński I., Pietrukowicz P., Kozłowski S., Skowron J. (2020). A terrestrial-mass rogue planet candidate detected in the shortest-timescale microlensing event. Astrophys. J. Lett..

[14] Fairén A.G., Gómez-Elvira J., Briones C., Prieto-Ballesteros O., Rodríguez-Manfredi J.A., López Heredero R., Belenguer T., Moral A.G., Moreno-Paz M., Parro V. (2020). The Complex Molecules Detector (CMOLD): A fluidic-based instrument suite to search for (bio)chemical complexity on Mars and icy moons. Astrobiology.

[15] Ruiz J., Fairén A.G. (2005). Seas under ice: Conditions for the stability of liquid-water oceans within icy worlds. Earth Moon Planets.

[16] Lingam M., Loeb A. (2019). Subsurface exolife. Int. J. Astrobiol..

[17] Lingam M., Loeb A. (2021). Life in the Cosmos: From Biosignatures to Technosignatures.

[18] Lingam M., Loeb A. (2020). Potential for liquid water biochemistry deep under the surface of Moon, Mars, and beyond. Astrophys. J. Lett..

[19] Irwin L.N., Schulze-Makuch D. (2003). Strategy for modeling putative ecosystems on Europa. Astrobiology.

[20] Abbot D., Switzer E. (2011). The Steppenwolf: A proposal for a habitable planet in interstellar space. Astrophys. J. Lett..

[21] Stevenson D. (1999). Life-Sustaining Planets in Interstellar Space?. Nature.

[22] Badescu V. (2011). Free-floating planets as potential seats for aqueous and non-aqueous life. Icarus.

[23] Shapiro R., Schulze-Makuch D. (2009). The search for alien life in our solar system: Strategies and priorities. Astrobiology.

[24] Lingam M., Loeb A. (2020). On the habitable lifetime of terrestrial worlds with high radionuclide abundances. Astrophys. J. Lett..

[25] Lyu Z., Shao N., Akinyemi T., Whitman W.B. (2018). Methanogenesis. Curr. Biol..

[26] Haldane J. (1929). The origin of life. Ration. Annu..

[27] Fox S.W., Dose K. (1977). Molecular Evolution and the Origin of Life.

[28] Miller S.L., Orgel L.E. (1974). The Origins of Life on the Earth.

[29] Chyba C.F., Thomas P.J., Brookshaw L., Sagan C. (1990). Cometary delivery of organic molecules to the early Earth. Science.

[30] Stevens T. (1997). Lithoautotrophy in the subsurface. FEMS Microbiol. Rev..

[31] D´Hondt S., Rutherford S., Spivack A.J. (2002). Metabolic activity of subsurface life in deep-sea sediments. Science.

[32] Stevens T.O., McKinley J.P. (1995). Lithoautotrophic microbial ecosystems in deep basalt aquifers. Science.

[33] Gold T. (1992). The deep, hot biosphere. Proc. Natl. Acad. Sci. USA.

[34] Sleep N., Zahnle K. (1998). Refugia from asteroid impacts on early Mars and the early Earth. J. Geophys. Res..

[35] González-Toril E., Martínez-Frías J., Gómez Gómez J.M., Rull F., Amils R. (2005). Iron meteorites can support the growth of acidophilic chemolithoautotrophic microorganisms. Astrobiology.

[36] Beatty J.T., Overmann J., Lince M.T., Manske A.K., Lang A.S., Blankenship R.E., Van Dover C.L., Martinson T.A., Plumley F.G. (2005). An obligately photosynthetic bacterial anaerobe from a deep-sea hydrothermal vent. Proc. Natl. Acad. Sci. USA.

[37] Muller A.W.J. (1985). Thermosynthesis by biomembranes: Energy gain from cyclic temperature changes. J. Theor. Biol..

[38] Muller A.W.J. (1995). Were the first organisms heat engines? A new model for biogenesis and the early evolution of biological energy conversion. Prog. Biophys. Molec. Biol..

[39] Muller A.W.J. (2003). Finding extraterrestrial organisms living on thermosynthesis. Astrobiology.

[40] Muller A.W.J., Schulze-Makuch D. (2006). Thermal energy and the origin of life. Orig. Life Evol. Biosph..

[41] Schulze-Makuch D., Irwin L.N. (2002). Energy cycling and hypothetical organisms in Europa’s ocean. Astrobiology.

[42] Madigan M.T., Martinko J.M., Parker J. (2000). Brock Biology of Microorganisms.

[43] Shihira-Ishikawa I., Nawata T. (1992). The structure and physiological properties of the cytoplasm in intact Valonia cell. Jpn. J. Phycol..

[44] Wilmer P., Stone G., Johnston I. (2000). Environmental Physiology of Animals.

[45] Heinz J., Krahn T., Schulze-Makuch D. (2020). A new record for microbial perchlorate tolerance: Fungal growth in NaClO_4_ Brines and its implications for putative life on Mars. Life.

[46] Breuer U., Harms H. (2006). *Debaryomyces hansenii*—An extremophilic yeast with biotechnological potential. Yeast.

[47] Irwin L.N., Schulze-Makuch D. (2020). The astrobiology of alien worlds: Known and unknown forms of life. Universe.

[48] Blakemore R.P. (1982). Magnetotactic bacteria. Annu. Rev. Microbiol..

[49] Gould J.L. (2018). Animal navigation: The evolution of magnetic orientation. Curr. Biol..

[50] Ibrahim A.I., Markwardt C.B., Swank J.H., Ransom S., Roberts M., Kaspi V., Woods P.M., Safi-Harb S., Balman S., Parke W.C. (2004). Discovery of a transient magnetar: XTE J1810-197. Astrophys. J. Lett..

[51] Debes J.H., Sigurdsson S. (2007). The survival rate of ejected terrestrial planets with moons. Astrophys. J..

[52] Sauvage J.F., Flinders A., Spivack A.J., Pockalny R., Dunlea A.G., Anderson C.H., Smith D.C., Murray R.W., D’Hondt S. (2021). The contribution of water radiolysis to marine sedimentary life. Nat. Commun..

[53] Lollar B.S., Heuer V.B., McDermott J., Tille S., Warr O., Moran J.J., Telling J., Hinrichs K.U. (2021). A window into the abiotic carbon cycle—Acetate and formate in fracture waters in 2.7 billion years-old host rocks of the Canadian Shield. Geochim. Cosmochim. Acta.

[54] Pirbadian S., Barchinger S.E., Leung K.M., Byun H.S., Jangir Y., Bouhenni R.A., Reed S.B., Romine M.F., Saffarini D.A., Shi L. (2014). *Shewanella oneidensis* MR-1 nanowires are outer membrane and periplasmic extensions of the extracellular electron transport components. Proc. Natl. Acad. Sci. USA.

[55] Rowe A.R., Chellamuthu P., Lam B., Okamoto A., Nealson K.H. (2015). Marine sediments microbes capable of electrode oxidations as a surrogate for lithotrophic insoluble substrate metabolism. Front. Microbiol..

[56] Strigari L., Barnabè M., Marshall P., Blandford R. (2012). Nomads of the Galaxy. Mon. Not. R. Astron. Soc..

[57] Meech K.J., Weryk R., Micheli M., Kleyna J.T., Hainaut O.R., Jedicke R., Wainscoat R.J., Chambers K.C., Keane J.V., Petric A. (2017). A brief visit from a red and extremely elongated interstellar asteroid. Nature.

[58] Opitom C., Fitzsimmons A., Jehin E., Moulane Y., Hainaut O., Meech K.J., Yang B., Snodgrass C., Micheli M., Keane J.V. (2019). 2I/Borisov: A C2-depleted interstellar comet. Astron. Astrophys..

[59] Bayaz R. (2012). Trillions of Rogue Planets with Life Roam Milky Way; Outnumber Stars by Factors of Thousands.

[60] Chyba C.F., Phillips C.B. (2001). Possible ecosystems and the search for life on Europa. Proc. Natl. Acad. Sci USA.

[61] Greenberg R., Geissler P., Tufts B.R., Hoppa G.V. (2000). Habitability of Europa´s crust: The role of tidal-tectonic processes. J. Geophys. Res. Planets.

[62] Roads E., Longton R.E., Convey P. (2014). Millennial timescale regeneration in a moss from Antarctica. Curr. Biol..

[63] Shmakova L., Malavin S., Iakovenko N., Vishnivetskaya T., Shain D., Plewka M., Rivkina E. (2021). A living bdelloid rotifer from 24,000-year-old Arctic permafrost. Curr. Biol..

[64] Shatilovich A.V., Tchesunov A.V., Neretina T.V., Grabarnik I.P., Gubin S.V., Vishnivetskaya T.A., Onstott T.C., Rivkina E.M. (2018). Viable nematodes from late Pleistocene permafrost of the Kolyma river lowland. Dokl. Biol. Sci..

[65] Yashina S., Gubin S., Maksimovich S., Yashina A., Gakhova E., Gilichinsky D. (2012). Regeneration of whole fertile plants from 30,000-y-old fruit tissue buried in Siberian permafrost. Proc. Natl. Acad. Sci. USA.

[66] Kirschvink J.L., Weiss B.P. (2002). Mars, panspermia, and the origin of life: Where did it all begin?. Palaeontol. Electron..

[67] Melosh H.J. (2003). Exchange of meteorites (and life?) between stellar systems. Astrobiology.

[68] Schulze-Makuch D., Irwin L.N., Fairén A.G. (2013). Drastic environmental change and its effects on a planetary biosphere. Icarus.

[69] Wickramasinghe N.C., Wallis J., Wallis D.H., Schild R.E., Gibson C.H. (2012). Life-bearing primordial planets in solar vicinity. Astrophys. Space Sci..

[70] Wallis M.K., Wickramasinghe N.C. (2004). Interstellar transfer of planetary microbiota. Mon. Not. R. Astron. Soc. Lett..

[71] Wickramasinghe J.T., Wickramasinghe N.-C., Napier W.M. (2010). Comets and the Origin of Life.

[72] Hartmann W.K., Davis D.R. (1975). Satellite-sized planetesimals and lunar origin. Icarus.

[73] Jacobsen S.B. (2005). The Hf-W isotopic system and the origin of the Earth and Moon. Annu. Rev. Earth Planet. Sci..

[74] Benz W., Slattery W.L., Cameron A.G.W. (1988). Collisional stripping of Mercury´s mantle. Icarus.

